# Supplementary material for: Hydrogen Gas-Grilling in Meat: Impact on Odor Profile and Contents of Polycyclic Aromatic Hydrocarbons and Volatile Organic Compounds
Source: Foods. 2024 Aug 2;13(15):2443. doi: 10.3390/foods13152443 (PMC11311495; doi:10.3390/foods13152443)
Supplement: Supplementary file 1 [file foods-13-02443-s001.zip › foods-3093250-supplementary.pdf]

Supplementary material

Table S1. Proximal composition of meat samples used in the experiment according to their fat level (very low and low). Data are expressed as percentage of raw product.

	mean	standard deviation	variation coefficient (%)
Moisture (%)			
very low-fat	75.17	0.66	0.89
low-fat	71.56	0.90	1.24
Protein (%)			
very low-fat	23.69	1.07	4.72
low-fat	22.92	0.70	3.05
Fat (%)			
very low-fat	0.41	0.21	41.18
low-fat	1.95	0.66	35.68
Ash (%)			
very low-fat	1.34	0.10	7.63
low-fat	1.29	0.14	11.02

ISO 1442:2023. Meat and meat products—Determination of moisture content (reference method); International Organization for Standardization: Geneva, Switzerland.

ISO 937:2023. Meat and meat products—Determination of nitrogen content (reference method); International Organization for Standardization: Geneva, Switzerland.

ISO 14143:1973. Meat and meat products—Determination of total fat content; International Organization for Standardization: Geneva, Switzerland.

ISO 936:1998. Meat and meat products—Determination of total ash; International Organization for Standardization: Geneva, Switzerland.

Table S2. Experimental conditions for quantifying PAHs by GC-MS according to Selective Ion Monitoring (SIM).

SIM-segment	Interval (min)	Compound	Quantifier	Qualifier	internal standard	Retention time (min)
1	6.0- 8.0	naphthalene	128	102	d_8-naphtalene	6.543
		d_8-naphtalene	136	-		
2	8.0- 9.8	acenaphthylene	152	151	d_{10}-acenaphtene	8.895
		acenaphthene	153	152	d_{10}-acenaphtene	9.119
		d_{10}-acenaphtene	164	-		
3	9.8- 11.5	fluorene	166	165	d_{10}-phenanthrene	10.137
4	11.5- 16.0	phenanthrene	178	176	d_{10}-phenanthrene	12.855
		anthracene	178	176	d_{10}-phenanthrene	12.973
		d_{10}-phenanthrene	188	-		
5	16.0- 20.0	fluoranthene	202	201	d_{10}-phenanthrene	16.673
		pyrene	202	200	d_{10}-phenanthrene	17.626
6	20.0- 24.0	benz[*a*]anthracene	228	226	d_{12}-chrysene	21.736
		chrysene	228	229	d_{12}-chrysene	22.012
		d_{12}-chrysene	240	-		
7	24.0- 30.0	benzo[*b*]fluoranthene	252	250	d_{12}-benzo[*a*]pyrene	25.883
		benzo[*k*]fluoranthene	252	250	d_{12}-benzo[*a*]pyrene	25.983
		benzo[*a*]pyrene	252	250	d_{12}-benzo[*a*]pyrene	27.735
		d_{12}-benzo[*a*]pyrene	264	-		
		d_{12}-perylene	264	-		
8	30.0- 37.2	benzo[*a,h*]anthracene	278	139	d_{12}-perylene	33.216
		indeno[1,2,3-*cd*]pyrene	276	274	d_{12}-perylene	33.283
		benzo[*ghi*]perylene	276	274	d_{12}-perylene	35.155

Table S3. PAHs used in this study and their absolute recoveries, and relative standard deviations (RSD).

	**1 ng/g**		**5 ng/g**		**50 ng/g**	
**Compound**	**recovery**	**% RSD**	**recovery**	**% RSD**	**recovery**	**% RSD**
acenaphthylene	113.45	9.3	103.90	4.8	101.66	0.1
naphthalene	90.17	44.6	120.34	12.2	98.75	3.2
pyrene	90.33	5.6	90.74	1.5	96.99	0.4
phenanthrene	110.18	14.5	102.12	3.4	101.99	1.6
fluoranthene	92.71	3.6	91.94	2.8	97.61	1.3
fluorene	93.01	14.1	98.32	2.8	106.50	0.3
acenaphthene	165.14	14.2	129.41	1.4	102.48	0.4
anthracene	108.14	2.0	106.64	5.2	115.01	0.3
chrysene	104.89	4.8	94.26	8.9	101.53	0.7
benzo[*a*]anthracene	102.87	5.4	104.05	0.7	112.16	1.0
benzo[*ghi*)perylene	66.14	8.0	61.43	1.0	69.35	1.2
indeno[1,2,3-*cd*]pyrene	71.84	0.1	65.67	0.0	78.65	0.6
benzo[*a*]pyrene	85.38	0.5	85.39	0.7	95.73	0.3
benzo[*b*]fluoranthene	86.65	0.1	89.96	3.0	100.53	0.2
benzo[*k*]fluoranthene	101.53	5.3	98.67	0.5	107.03	0.1
dibenzo[*a,h*]anthracene	75.31	3.3	75.74	4.1	86.42	0.6
**Average**	**97.36**	**8.4**	**94.91**	**3.31**	**98.27**	**0.8**

Table S4. VOCs that were detected but not quantified due to their concentration being below the limit.

Aldehydes	*Alcohols*
2-heptanal	1-octanol
2-hexenal	2-pentyn-1-ol
2-butenal	1-hexanol
2,4-heptadienal	3-hexen-1-ol
2-methyl-2-butenal	*Ketones*
2-ethyl-*trans*-2-butenal	2,3-octanedione
Alkanes	6-methyl-2-heptanone
Octane	2-methyl-cyclopentanone
2,4-octadiene	2-octanone
4-methyl-2-nonene	3-hexanone
1-octene	3-cyclohepten-1-one
undecane	3-ethylcyclopentanone
methyl cyclopentane	*Furans*
3-methylnonane	2-penthylfuran
1,3-pentadiene	2-methylfuran
propyl cyclopentane	2-buthylfuran
(E) 2-octene	*Others*
1-acetylcyclo hexene	xylene
3-ethyl-3-methyl heptane	ethyl benzene
	ethyl acetate

Table S5. Odor profile of meat samples according to fat content (very low and low) and gas-grilling (butane and hydrogen). Data are expressed as logarithm of absolute values.

	very low-fat horse meat		low-fat horse meat	
**Sensor**	**butane grill**	**hydrogen grill**	**butane grill**	**hydrogen grill**
LY2/LG	12.22 ± 0.05a	12.26 ± 0.02b	12.23 ± 0.05ab	12.28 ± 0.06b
LY2/G	11.16 ± 0.17a	11.28 ± 0.15ab	11.29 ± 0.13b	11.17 ± 0.12ab
LY2/AA	10.99 ± 0.29a	11.15 ± 0.17ab	11.19 ± 0.24b	11.08 ± 0.17ab
LY2/Gh	11.08 ± 0.10	11.14 ± 0.15	11.14 ± 0.16	11.06 ± 0.08
LY2/gCTI	11.09 ± 0.19	11.17 ± 0.14	11.21 ± 0.16	11.13 ± 0.11
LY2/gCT	12.23 ± 0.16	12.20 ± 0.22	12.32 ± 0.27	12.16 ± 0.19
T30/1	-0.44 ± 0.06a	-0.35 ± 0.04b	-0.38 ± 0.09b	-0.35 ± 0.04b
P10/1	-0.60 ± 0.05a	-0.49 ± 0.04b	-0.53 ± 0.08b	-0.50 ± 0.05b
P10/2	-0.78 ± 0.08a	-0.62 ± 0.06b	-0.68 ± 0.12b	-0.63 ± 0.06b
P40/1	-0.53 ± 0.07a	-0.41 ± 0.05b	-0.44 ± 0.10b	-0.42 ± 0.07b
T70/2	-0.52 ± 0.06a	-0.38 ± 0.05b	-0.44 ± 0.08b	-0.38 ± 0.07b
PA/2	-0.52 ± 0.10a	-0.33 ± 0.06b	-0.40 ± 0.13b	-0.34 ± 0.07b
P30/1	-0.42 ± 0.07a	-0.32 ± 0.04b	-0.35 ± 0.09b	-0.32 ± 0.05b
P40/2	-0.57 ± 0.03a	-0.54 ± 0.03ab	-0.55 ± 0.05b	-0.53 ± 0.04b
P30/2	12.91 ± 0.05	12.85 ± 0.13	12.87 ± 0.13	12.83 ± 0.11
T40/2	-0.91 ± 0.05	-0.88 ± 0.06	-0.86 ± 0.11	-0.91 ± 0.08
T40/1	-0.68 ± 0.05a	-0.60 ± 0.05ab	-0.62 ± 0.09b	-0.61 ± 0.07b
TA/2	-0.56 ± 0.04a	-0.49 ± 0.04b	-0.51 ± 0.08ab	-0.50 ± 0.07ab

Different letters (a,b) in the same row are significantly different ($p < 0.05$).
